# Cerebellar granule neuron progenitors are the source of Hk2 in the postnatal cerebellum

**DOI:** 10.1186/2049-3002-1-15

**Published:** 2013-06-11

**Authors:** Timothy R Gershon, Andrew J Crowther, Hedi Liu, C Ryan Miller, Mohanish Deshmukh

**Affiliations:** 1 Department of Neurology, University of North Carolina, Chapel Hill, NC 27599, USA; 2 Neuroscience Center, University of North Carolina, Chapel Hill, NC 27599, USA; 3 Lineberger Comprehensive Cancer Center, University of North Carolina, Chapel Hill, NC 27599, USA; 4 Department of Pathology, Division of Neuropathology, University of North Carolina, Chapel Hill, NC 27599, USA; 5 Department of Cell and Developmental Biology, University of North Carolina, Chapel Hill, NC 27599, USA; 6 UNC School of Medicine, 170 Manning Drive CB7025, Chapel Hill, NC 27599, USA

## Correspondence

A response to Leprince: **The role of Bergmann glial cells in cerebellar development.***Cancer & Metabolism* 2013, **1**:14

We recently demonstrated that developmentally regulated aerobic glycolysis is integral to the normal process of postnatal neurogenesis and becomes co-opted in medulloblastoma. In our work, we concluded that Hexokinase 2 (Hk2), which we found to be required for Shh-induced aerobic glycolysis, was expressed specifically by cerebellar granule neuron progenitors (CGNPs). We observed altered migration of CGNPs in hGFAP-cre;Hk2^f/f^ mice and attributed this aspect of the phenotype to premature differentiation of CGNPs caused by loss of aerobic glycolysis. In response to our work, LePrince draws attention to the role of Bergmann glia in cerebellar development.

LePrince raises the important point that cerebellar granule neurons (CGNPs) do not develop in isolation but rather interact critically with the Bergmann glia. The Bergmann glia establish a radial scaffold on which the CGNPs migrate from the external to the internal granule cell layer [1,2]. Our data are consistent with the possibility that conditional deletion of Hk2 disrupts the interaction through which CGNPs are guided in their migration by the processes of the Bergmann glia. The idea that cellular metabolic patterns may modulate developmental signaling between CGNPs and glia fits well with our finding that developmental signaling between Purkinje cells and CGNPs, mediated by Shh, regulates CGNP metabolism.

**Figure 1:**
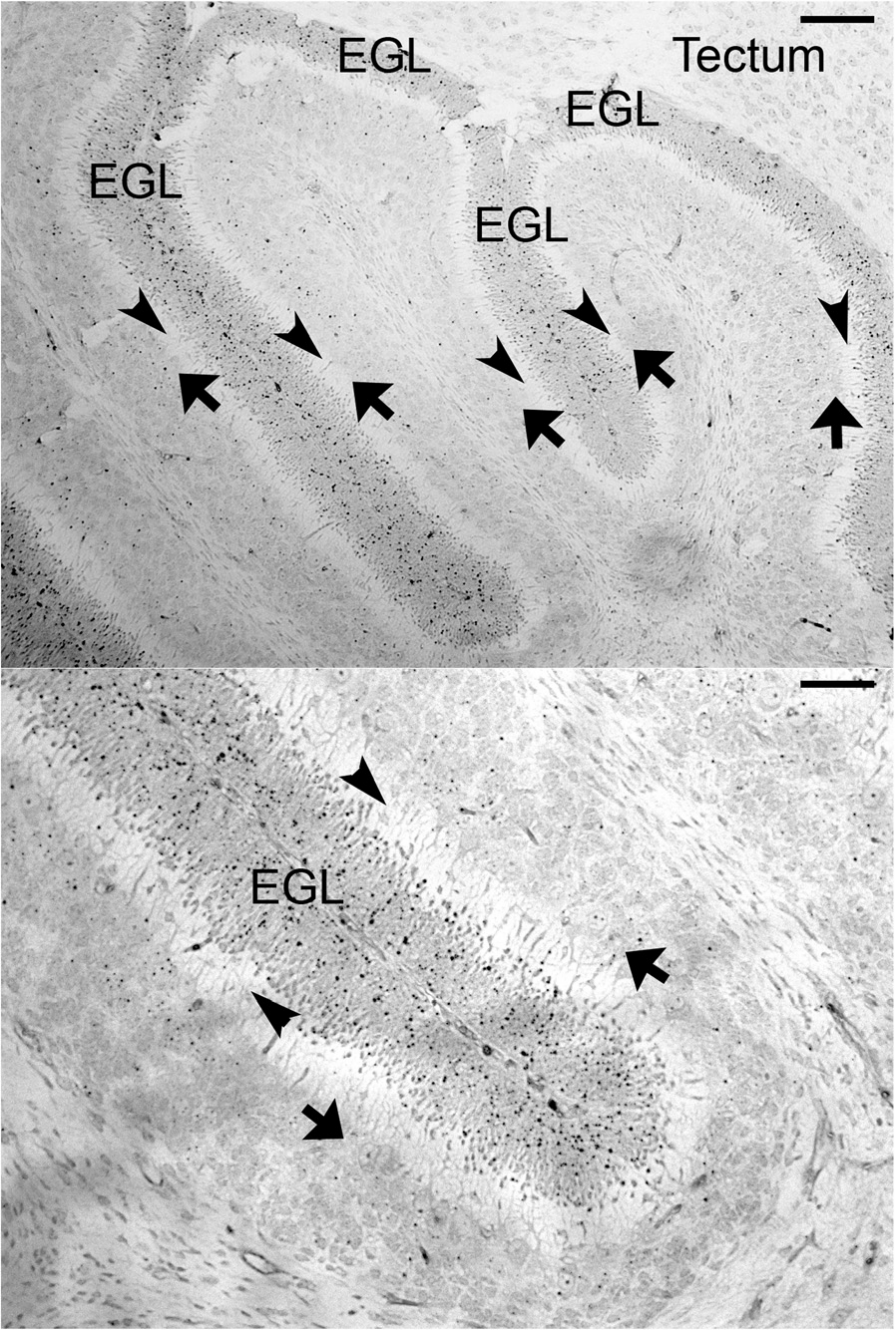
**Hk2 mRNA is expressed by CGNPs.** In low magnification (upper panel) and higher magnification (lower panel) views, *in situ* hybridization demonstrates Hk2 mRNA in the external granule layer (EGL), which is populated by CGNPs. Bergmann glia have cell bodies in the Purkinje cell layer (arrows) and project radial processes through the molecular layer (arrowheads). *In situ* hybridization did not demonstrate Hk2 mRNA in these regions. Bars represent 100 μm in the upper panel and 50 μm in the lower panel.

In addition to underscoring the importance of Bergmann glia in cerebellar histogenesis, LePrince questions whether the Bergmann glia are in fact the source of Hk2. While we feel that we correctly labeled the EGL in Figure 3e of our paper [3], we recently used *in situ* hybridization, which allowed us to use paraffin sections, to resolve the postnatal expression pattern of Hk2 in greater detail. We examined paraffin sections of cerebellum from P9 mice, where the EGL is thinner and the molecular layer is thicker than in the P7 mice presented in Fig. 3e. Our results confirm that the CGNPs in the EGL are the predominant source of Hk2 mRNA in the postnatal cerebellum (Figure 1). While we recognize that hGFAP-cre would drive recombination of the floxed Hk2 alleles in Bergmann glia, as well as in CGNPs, such recombination is not likely to alter the metabolism of these glia as they do not seem to express significant Hk2 mRNA. In contrast, the CGNPs demonstrate robust expression of Hk2, and are thus more likely to be critically affected by Hk2 deletion.

We appreciate the emphasis that LePrince places on the role of Bergmann glia in directing CGNP migration. Our data identify the CGNPs as the principal cells actuating Hk2-mediated aerobic glycolysis. The possibility that loss of aerobic glycolysis disrupts the interaction of CGNPs with the Bergmann glia deserves further investigation.

## Methods

Mice were handled Mice were handled in compliance with the guidelines of the University of North Carolina Animal Care and Use Committee (IACUC#10-126). Brains from P9 wild type BL6 pups were fixed in 4% formaldehyde, embedded in paraffin and 5 mm sagittal sections were cut and placed on glass slides. Slides were processed and stained using the RNAscope 2.0 kit (cat#310095, Advanced Cell Diagnostics, Hayward, CA) according to manufacturer instructions, with the insertion of a 10 minute DNAse treatment step at 40°C before hybridization, using RNAse-free DNAse 1u/μl in 1xDNAse buffer (Cat# M6101, Promega, Madison, WI).

## Competing interests

The authors declare that they have no competing interests.

## Authors’ contributions

TRG designed the experiments, generated the figures and drafted the manuscript. AJC performed all breeding and genotyping, and reviewed the manuscript and figures. HL performed the in situ hybridizations and reviewed the manuscript and figures. CRM provided neuropathology expertise and reviewed the manuscript and figures. MD provided key input in the experimental design and drafting of the manuscript and reviewed the manuscript and figures. All authors have read and approved the final manuscript.
